# Molecular Mechanisms of Radioiodine Refractoriness in Differentiated Thyroid Cancer: Focus on Sodium/Iodide Symporter Dysregulation

**DOI:** 10.3390/cimb48040362

**Published:** 2026-03-31

**Authors:** Vladimir D. Rokashkov, Liudmila V. Spirina, Natalya V. Tarasenko, Svetlana Yu. Chizhevskaya

**Affiliations:** 1 Biochemistry and Molecular Biology Division, Siberian State Medical University, 2 Moskovsky Trakt, Tomsk 634050, Russia; vovalol31@gmail.com (V.D.R.); natvt2003@mail.ru (N.V.T.); sch@oncology.tomsjk.ru (S.Y.C.); 2 Cancer Research Institute, Tomsk National Research Medical Center of the Russian Academy of Sciences, 5 Kooperativny Street, Tomsk 634050, Russia

**Keywords:** thyroid cancer, NIS (sodium/iodide symporter), microRNAs, radioiodine refractoriness, biomarkers

## Abstract

The sodium/iodide symporter (NIS/SLC5A5) is a major determinant of radioiodine therapy efficacy in differentiated thyroid cancer (DTC). This narrative review examines the molecular mechanisms underlying NIS dysregulation and radioiodine refractoriness in DTC. Reduced NIS expression or function in radioiodine-refractory DTC is associated with multiple mechanisms, including transcriptional suppression linked to MAPK/ERK and PI3K/AKT pathway activation and disruption of thyroid differentiation programs; epigenetic silencing involving SLC5A5 regulatory regions; impaired protein trafficking and membrane localization; and post-transcriptional regulation by microRNAs such as miR-221-3p, miR-222-3p, miR-146b-3p, and miR-204-5p. Genetic alterations including BRAF V600E and TERT promoter mutations are associated with dedifferentiated tumor phenotypes and poor radioiodine response. Redifferentiation approaches using MAPK pathway inhibitors such as selumetinib and dabrafenib can restore iodine uptake in selected patients, although the overall clinical applicability of these strategies remains under evaluation. A better understanding of these mechanisms may support improved biologic stratification and more selective therapeutic decision-making in radioiodine-refractory DTC.

## 1. Introduction

Differentiated thyroid cancer (DTC) accounts for more than 90% of all thyroid malignancies and is characterized by a favorable prognosis with ten-year survival exceeding 90% [1,2,3]. An important component of treatment for appropriately selected patients is radioiodine therapy (RAI) with I-131, which depends on the preservation of functional activity of the sodium/iodide symporter (NIS/SLC5A5)—a specialized membrane protein that mediates active iodide transport and, in normal thyroid tissue, creates an iodide concentration gradient of 20–40:1 between the thyrocyte and serum [4,5,6]. In metastatic DTC, the clinical benefit of RAI is determined by the ability of tumor foci to accumulate radioiodine [2,3].

However, a substantial subset of patients, particularly those with metastatic disease, develop radioiodine refractoriness—the loss of the tumor’s ability to accumulate therapeutically significant doses of I-131, which is associated with a marked deterioration in prognosis and a life expectancy of about 3–5 years in advanced disease [2,3,7]. Molecular mechanisms of radioiodine refractoriness include decreased NIS expression and impaired membrane localization [2,8,9,10,11,12]. Loss of thyroid-specific gene expression and disruption of transcription factor-dependent differentiation programs further reduce iodine avidity [9,10,13,14]. These changes are driven in part by oncogenic signaling through the BRAF/MAPK/ERK and PI3K/AKT pathways and occur within broader tumor dedifferentiation programs [2,9,10,11,12,15,16].

In recent years, compelling evidence has accumulated regarding the role of microRNAs in DTC biology and in pathways associated with radioiodine response [17,18]. MicroRNAs appear to influence iodide-handling and tumor differentiation predominantly through indirect regulatory networks. In particular, miR-221-3p and miR-222-3p are consistently overexpressed in papillary thyroid carcinoma and promote proliferation through suppression of p27Kip1, while inflammatory signaling such as HMGB1 can further enhance their expression [19,20]. miR-146b-3p has been implicated in repression of the PAX8/NIS axis and reduced radioiodide sensitivity [18]. In contrast, miR-204-5p exerts tumor-suppressive effects in thyroid cancer through targets including HMGA2 and IGFBP5 [21,22]. This review focuses on these microRNAs because they are among the most extensively studied in thyroid cancer, have substantial experimental support for their involvement in proliferation and differentiation-associated pathways [18,19,20,21,22], and form a combined expression profile that has been proposed as a predictor of tumor progression after postoperative RAI ablation [23]. The microRNA expression profile characterized by high miR-221-3p/miR-222-3p and low miR-204-5p showed strong prognostic performance for progression-free survival in DTC cohorts, including patients treated with postoperative RAI [23]. Approximately 30–40% of differentiated thyroid cancers do not have identifiable BRAF, RAS mutations or RET/NTRK fusions [15], making microRNA dysregulation a key but insufficiently studied mechanism that requires the development of standardized clinical biomarker panels.

The aim of this review is to summarize current knowledge on the role of the sodium/iodide symporter and selected microRNAs in the molecular pathogenesis of radioiodine refractoriness in differentiated thyroid cancer, and to discuss their possible relevance as biologic markers and therapeutic targets. The review covers the iodide uptake and metabolism system in thyrocytes (NIS, TPO, TG, and the transcription factors PAX8, NKX2-1, and FOXE1), current clinical criteria for radioiodine refractoriness, genetic and epigenetic mechanisms of impaired iodine handling, the role of microRNAs in differentiation-associated pathways, and the prognostic significance of selected biomarker panels in the context of radioiodine therapy.

A narrative literature search was conducted in PubMed/MEDLINE, Web of Science, and Scopus databases covering publications from 1996 to March 2025. Search terms included combinations of “sodium iodide symporter”, “NIS”, “SLC5A5”, “radioiodine refractory”, “radioiodine resistance”, “differentiated thyroid cancer”, “thyroid dedifferentiation”, “miR-221-3p”, “miR-222-3p”, “miR-204-5”, “miR-146b-3p”, “BRAF V600E”, “TERT promoter”, “redifferentiation therapy” and related terms. Additional references were identified through manual screening of reference lists of relevant reviews and original articles. Priority was given to original research articles, systematic reviews, and clinical guidelines published in peer-reviewed journals.

## 2. Mechanisms of Iodide Uptake and Transformation in Thyroid Cells

### 2.1. NIS Structure, Function, and Regulation

The sodium/iodide symporter (NIS, Na/I-symporter, SLC5A5) is an integral membrane glycoprotein that provides active iodide transport across the basolateral membrane of thyrocytes [4]. The SLC5A5 gene encoding NIS is located on chromosome 19p13.2-p12, spans approximately 23 kilobase pairs, and consists of 15 exons interrupted by 14 introns [5]. The human NIS protein contains 643 amino acid residues and has a molecular mass of approximately 70–90 kDa depending on the degree of glycosylation [5,24,25].

Structural analysis of NIS revealed 13 transmembrane segments with an extracellular NH2 terminus and an intracellular COOH terminus [4,26]. NIS functions as an electrogenic symporter, co-transporting two sodium ions and one iodide ion (stoichiometry Na^+^:I^−^ = 2:1) across the plasma membrane [5,25,26]. The driving force for iodide accumulation against the concentration gradient is the sodium electrochemical gradient maintained by Na^+^/K^+^-ATPase [4,5,25,26].

The functional activity of NIS largely depends on its correct localization at the basolateral membrane of thyrocytes [25]. Post-translational modifications, including N-glycosylation at three sites (Asn225, Asn489, and Asn502), participate in protein maturation and modulate transport efficiency [27,28]. However, available mechanistic studies indicate that glycosylation is not essential for plasma membrane targeting, and substantial residual transport activity may persist even in the absence of glycosylation [27].

NIS expression and functional activity are under the strict control of thyroid-stimulating hormone (TSH) [29]. TSH binding to the TSH receptor (TSHR) on the basolateral membrane of thyrocytes activates adenylate cyclase through G-proteins, leading to increased intracellular cyclic adenosine monophosphate (cAMP) levels and activation of protein kinase A (PKA) [29,30]. This signaling pathway induces SLC5A5 gene transcription, promotes NIS biosynthesis and targeting to the plasma membrane, and modulates its phosphorylation pattern and stability [30,31]. Besides TSH, NIS expression can also be modulated in experimental systems by factors such as IGF-1, thyroglobulin, estradiol, cytokines, and retinoic acid [8].

The clinical significance of NIS is determined by its key role in the uptake of radioactive iodine-131 (131I) used for the diagnosis and treatment of thyroid cancer [8]. Decreased NIS expression or impaired membrane localization is one of the main mechanisms of radioiodine resistance development in thyroid tumors [1,8].

### 2.2. Role of NIS in Radioactive Iodine-131 Uptake

The ability of NIS to transport radioactive iodine isotopes, primarily iodine-131 (131I), is the basis for diagnosis and treatment of DTC [4,5]. The mechanism of 131I uptake is identical to that of stable iodine-127 transport: NIS does not distinguish between iodine isotopes and co-transports them with sodium ions in a 2:1 ratio across the basolateral membrane of thyrocytes [5]. This feature allows 131I to be used both for visualization of thyroid tissue and its metastases by scintigraphy and for therapeutic destruction of tumor cells through local β- and γ-radiation [6,8].

Radioiodine therapy (RAI) remains an important postoperative treatment option for selected patients with differentiated thyroid cancer after thyroidectomy [32,33]. After oral administration, 131I actively accumulates in residual thyroid tissue and tumor metastases expressing functional NIS on the membrane [8]. Beta emission from 131I mediates local cytotoxicity in NIS-expressing tissue, whereas gamma emission enables scintigraphic visualization of isotope distribution and assessment of radioiodine uptake by tumor tissue [6,27,34].

Effective use of iodide in the thyroid depends not only on basolateral uptake, but also on apical transport and organification. After entering the thyrocyte, iodide is transported across the apical membrane into the follicular lumen predominantly via pendrin, where thyroid peroxidase uses hydrogen peroxide generated by dual oxidases to oxidize and organify iodide on thyroglobulin [35,36]. Downstream thyroid hormone activation and inactivation are then modulated by iodothyronine deiodinases, underscoring that clinically meaningful radioiodine avidity reflects preservation of a broader iodine-handling program rather than NIS expression alone [37].

RAI effectiveness directly depends on the level of NIS expression and functional activity in tumor cells [4,25]. Membrane localization of NIS, rather than intracellular immunoreactivity alone, is the critical prerequisite for effective iodide uptake [38]. Conversely, decreased NIS expression or its intracellular sequestration lead to insufficient radioiodine uptake and development of radioiodine resistance [1,25,38].

In clinical practice, RAI effectiveness is optimized by TSH stimulation [33]. Elevation of TSH levels (endogenous, achieved by withdrawal of thyroxine suppressive therapy, or exogenous recombinant human TSH) stimulates NIS expression and enhances 131I uptake in both residual thyroid tissue and metastases of differentiated DTC [33]. TSH levels > 30 mIU/L are generally considered optimal for RAI [32,33].

Clinically, 131I uptake is assessed using diagnostic or post-therapeutic whole-body scintigraphy [32,34]. Absence of radioiodine accumulation in metastatic foci despite adequate TSH stimulation indicates loss of functional radioiodine avidity and is one of the key clinical features of radioiodine-refractory disease [32,34]. Besides NIS expression, 131I uptake efficiency is affected by iodide availability in blood, competitive inhibition by perchlorate or thiocyanate, tumor differentiation degree, and NIS post-translational regulation status [4,5,8].

## 3. Definition and Mechanisms of Resistance to Radioiodine Therapy

### 3.1. ATA 2025 Criteria for Radioiodine Refractoriness

Radioiodine-refractory (RR) differentiated thyroid cancer represents a clinically significant subgroup of tumors characterized by the inability to accumulate radioactive iodine in therapeutically meaningful concentrations, rendering traditional radioiodine therapy ineffective [2,10,32]. According to the 2025 American Thyroid Association Guidelines (ATA 2025), radioiodine refractoriness cannot be diagnosed in patients who have not received an ablative or treatment dose of RAI, and is defined as a condition in which tumor tissue loses the ability to concentrate I-131 or demonstrates disease progression despite adequate radioiodine therapy [32]. This patient category has a substantially worse prognosis than radioiodine-sensitive metastatic disease; reviews and consensus documents describe 10-year survival below 10–15% in many RAIR series and note that some patients with progressive metastatic disease die within 3–5 years [2,3,10].

The ATA 2025 guidelines define strong criteria suggesting iodine-refractory DTC [32]: (i) absence of 131I uptake on post-therapy scan in the setting of confirmed disease visible on structural imaging (CT, MRI) or 18F-FDG-PET (when tumor tissue fails to concentrate the radiopharmaceutical on post-therapy scintigraphy despite adequate patient preparation; this may occur at initial treatment of metastatic DTC or at subsequent RAI administration); and/or (ii) disease progression fewer than 6 months after a treatment-appropriate administration of therapeutic RAI that demonstrated uptake on post-therapy scans (demonstrated by growth of metastases or appearance of new foci on structural imaging). When a patient does not have uptake on post-therapy scans in the setting of structurally apparent disease and/or when there is progression fewer than 6 months after treatment, such patient is unlikely to receive benefit from additional RAI administration [32].

The ATA 2025 guidelines also describe supplemental criteria suggesting less RAI sensitivity [32]: (A) no uptake present on diagnostic 123I or 131I whole-body scan in the presence of otherwise detectable disease (this criterion predicts less favorable response to RAI, but some patients may have a positive post-therapy scan and derive clinical benefit); (B) heterogeneous I-131 uptake, when uptake is present in some but not all tumor foci on post-therapy whole-body scan (indicating biological tumor heterogeneity, with some metastatic foci showing preserved radiopharmaceutical accumulation while others exhibit no uptake; this suggests that multimodal treatment approach may be appropriate, as RAI alone is not adequate for this subgroup). Achievement of cumulative I-131 activity ≥ 600 mCi (22 GBq) is associated with high risk of distant toxic effects (secondary hematological malignancies, pulmonary fibrosis, xerostomia) and serves as an important consideration in treatment decisions, but does not by itself define radioiodine refractoriness [3,32]. Response to RAI can be heterogeneous, and RAIR criteria should be used to risk-stratify patients about the likelihood that their tumors will respond to RAI rather than serve as absolute criteria mandating treatment decisions [32].

It is important to distinguish between “radioiodine resistance” and “radioiodine refractoriness” [2,3]. Radioiodine resistance is a broader term describing a biological characteristic of tumor cells, namely the reduced ability to accumulate or retain radioactive iodine because of molecular abnormalities such as decreased NIS expression and dedifferentiation. Radioiodine refractoriness is a clinical diagnosis established on the basis of the above ATA 2025 criteria and used to guide further patient management [3,32].

Molecular mechanisms of radioiodine refractoriness development include decreased or lost expression of iodine-handling proteins (NIS, TPO, TG), loss of NIS membrane localization, dedifferentiation with suppression of thyroid-specific transcriptional programs, activation of oncogenic signaling pathways such as MAPK and PI3K/AKT, and epigenetic silencing of iodide-metabolism genes [2,10]. Understanding these mechanisms is critical for developing redifferentiation therapy strategies aimed at restoring tumor cells’ ability to accumulate radioactive iodine [2,10].

Clinical management of patients with radioiodine-refractory differentiated thyroid cancer requires a multidisciplinary approach and may include observation of stable disease, local treatment methods (surgery, radiation therapy, radiofrequency ablation), systemic targeted therapy with tyrosine kinase inhibitors (sorafenib, lenvatinib), or experimental redifferentiation therapy protocols [2,3,7,32]. Treatment choice is determined by disease progression rate, location and volume of metastatic involvement, as well as patient’s general condition and presence of symptoms [7,32].

### 3.2. Prevalence and Prognostic Significance of Radioiodine Therapy Resistance

Radioiodine-refractory differentiated thyroid cancer is uncommon in the overall DTC population but becomes a major clinical problem in patients with distant metastases [2,3,39]. Distant metastases occur in less than 10% of patients with papillary and follicular thyroid carcinoma, and only about two-thirds of patients with metastatic disease show substantial radioiodine uptake at presentation [3,39]. Consequently, a substantial subset of metastatic patients either never demonstrates clinically meaningful iodine avidity or loses it over time, which is the population that defines RAIR disease in clinical practice [2,3,32].

The likelihood of radioiodine refractoriness is influenced by tumor biology [9,15]. Papillary thyroid carcinomas harboring BRAF V600E show reduced expression of key iodide-handling genes, including NIS, AIT-B, Tg, and TPO, and the NIS protein that is expressed is frequently retained in the cytoplasm rather than correctly localized to the membrane [9]. Consistently, molecular management studies associate BRAF-mutant PTC with loss of radioiodine avidity in recurrent disease [15]. However, RAIR is not confined to BRAF-mutant tumors and can also arise in tumors driven by other dedifferentiation mechanisms, including alterations affecting RAS- and PI3K/AKT-related pathways [15].

The clinical and prognostic significance of radioiodine refractoriness is substantial [2,3,39]. Reviews of progressive RAIR metastatic DTC describe median survival on the order of 3–5 years and 10-year survival often below 10–15% [2,3]. By contrast, outcomes are markedly better in patients with iodine-avid, treatment-responsive metastases: 10-year overall survival after initiation of 131I treatment was reported as 92% in patients who achieved negative imaging studies and 19% in those who did not [39]. In another series of patients presenting with distant metastases, age older than 45 years, follicular histology, and extrapulmonary metastases were associated with lower disease-specific survival [40].

Clinical and pathological features associated with poorer radioiodine response or worse outcome include older age, less differentiated histology, more extensive metastatic burden, extrapulmonary disease, low or absent 131I uptake, and high 18F-FDG uptake [32,39,40]. Molecular alterations such as BRAF V600E are also linked to reduced expression of iodide-handling genes and lower radioiodine avidity [9,15].

Given the pronounced effect of radioiodine refractoriness on prognosis, there is a strong need for development and validation of predictive biomarkers that could identify patients at high risk of RAIR disease at earlier stages [2,3,41]. Candidate predictors include molecular alterations affecting differentiation and iodide handling, serum thyroglobulin dynamics, post-therapy uptake patterns, and imaging parameters such as PET/CT findings; multivariate models integrating demographic, clinicopathological, serological, and imaging data have also shown potential predictive value [2,41]. Identification of such patients could help optimize treatment selection, timing of local or systemic therapy, and enrollment in clinical trials of redifferentiation strategies [2,3].

Furthermore, understanding the molecular mechanisms underlying radioiodine refractoriness and their relationship to clinical outcomes opens prospects for more personalized treatment approaches [2,10,41]. Integration of tumor molecular profiling into clinical practice may facilitate patient stratification by RAIR risk and support adaptation of therapeutic strategies to individual tumor biology [2,10,41].

## 4. Molecular Mechanisms of Radioiodine Resistance

### 4.1. Decreased NIS Expression and Impaired Localization

Decreased sodium/iodide symporter (NIS) expression and impaired subcellular localization represent fundamental mechanisms of radioiodine refractoriness development in differentiated thyroid cancer (DTC). Loss of NIS functional activity can occur at various levels: from transcriptional suppression of the SLC5A5 gene to impairments in post-translational modification and intracellular protein transport. Understanding these mechanisms is critically important for developing strategies to restore iodine uptake and overcome therapeutic resistance [2,4,8,10].

#### 4.1.1. Transcriptional Suppression of NIS Expression

Decreased SLC5A5 gene mRNA levels are among the most common molecular abnormalities in radioiodine-refractory DTC. Available clinical and experimental data consistently associate reduced NIS expression with impaired iodine avidity, although refractoriness also depends on membrane targeting and the broader degree of thyroid tumor differentiation [8,9,10]. A key contribution to this process comes from disruption of transcriptional programs that normally maintain thyroid-specific gene expression.

PAX8 and NKX2-1 (TTF-1) are key regulators of thyroid differentiation and participate in control of SLC5A5 transcription [13,14,42]. Experimental studies show that Pax8 binds the NIS upstream enhancer and is required for thyroid-selective, cAMP-responsive activation of NIS transcription, while the broader thyroid differentiation program also depends on the coordinated action of thyroid-specific transcription factors including NKX2-1 [13,42,43]. At the same time, the attached sources do not support reducing NIS loss in all radioiodine-refractory tumors to isolated downregulation of these two factors alone; rather, NIS repression appears to occur within a wider dedifferentiation program affecting multiple thyroid-specific genes [10,14].

Oncogenic transformation in DTC is accompanied by activation of key MAPK and PI3K/AKT signaling cascades, which exert multiple inhibitory effects on NIS expression [9,10,44]. The BRAFV600E mutation, occurring in approximately 45% of papillary thyroid carcinomas, leads to constitutive activation of the MAPK/ERK pathway [9,10,12]. In BRAFV600E-driven thyroid models, NIS repression is mediated in part by induction of transforming growth factor beta (TGF-beta) secretion, which suppresses the SLC5A5 promoter through SMAD-dependent signaling; this TGF-beta axis also cooperates with MEK-ERK signaling to promote invasion and epithelial–mesenchymal transition [44]. PI3K/AKT pathway activation is likewise discussed as part of the dedifferentiation network associated with loss of thyroid-specific gene expression and reduced radioiodine avidity [2,10].

Overall, NIS transcriptional suppression tends to become more pronounced as tumors dedifferentiate and progress, which helps explain the clinical observation that some initially iodine-avid tumors later lose responsiveness to radioiodine therapy [9,10].

#### 4.1.2. Post-Translational Modifications and NIS Membrane Localization

Even with preserved SLC5A5 gene transcription, NIS functional activity can be impaired at the post-translational level. Correct NIS protein membrane localization on the basolateral surface of thyrocytes is absolutely necessary for iodide transport [4,5,28]. NIS is subject to post-translational regulation, including glycosylation, phosphorylation, and vesicular trafficking, all of which can influence the efficiency of plasma membrane delivery.

NIS N-glycosylation participates in protein maturation, but available mechanistic studies also show that glycosylation is not strictly required for NIS stability, plasma membrane targeting, or transport activity [27,28]. NIS contains three N-glycosylation sites located in extracellular domains [27]. Studies of trafficking-defective NIS mutants further show that impaired maturation can be associated with intracellular retention and failure to reach the cell surface, although available data do not support attributing this effect to loss of glycosylation per se [28]. In thyroid cancer, these data support impaired maturation and trafficking as plausible contributors to functional NIS loss, but the attached sources do not justify presenting abnormal glycosylation as a universally established mechanism in all radioiodine-refractory tumors [8,10].

Correct NIS membrane localization is also regulated by TSH-dependent post-transcriptional mechanisms. In FRTL-5 cells, TSH induces de novo NIS biosynthesis, modulates its phosphorylation pattern, and is required for targeting or retention of NIS at the plasma membrane; after TSH withdrawal, NIS redistributes to intracellular compartments despite persistence of transporter activity in membrane vesicles [30,31]. These observations support the concept that impaired trafficking can reduce iodide uptake even when NIS protein is still present. A similar dissociation between NIS expression and functional membrane localization has been documented in thyroid tumors, where immunohistochemistry often shows predominantly cytoplasmic rather than membranous staining [9,38].

Recent proteomic studies have identified novel NIS-interacting proteins that regulate its vesicular trafficking. ADP-ribosylation factor 4 (ARF4) enhances NIS trafficking from the Golgi to the plasma membrane, whereas valosin-containing protein (VCP)—a principal component of endoplasmic reticulum-associated degradation—governs NIS proteolysis [45]. VCP expression is particularly induced in aggressive thyroid cancers and in patients with poorer outcomes following RAI treatment. Notably, FDA-approved VCP inhibitors abrogated VCP-mediated repression of NIS function, resulting in significantly increased NIS at the cell surface and markedly increased RAI uptake in mouse and human thyroid models [45]. These findings highlight the therapeutic potential of targeting NIS trafficking pathways to restore radioiodine sensitivity.

### 4.2. Intracellular NIS Sequestration and Its Clinical Significance

The phenomenon of intracellular (cytoplasmic) NIS sequestration represents a mechanism of iodine uptake loss in which NIS protein may be detectable in tumor cells but is not efficiently delivered to, or retained at, the plasma membrane [8,10,25,38]. Classic immunohistochemical studies in thyroid carcinomas reported predominant intracellular staining compared with the membranous pattern seen in normal thyrocytes [38,46]. In a larger immunohistochemical series, 73% of thyroid cancers were NIS-positive by immunohistochemistry, but most positive tumors showed strong intracellular staining without clear plasma membrane localization, whereas only some papillary carcinomas displayed a membranous pattern [38]. These observations support the concept that preserved immunoreactivity does not necessarily indicate preserved iodide-transport function.

Mechanisms leading to cytoplasmic NIS sequestration in DTC include impaired vesicular transport and disturbed post-translational trafficking, together with altered interactions with proteins that regulate membrane delivery and turnover [8,10,25]. Recent work identified specific NIS-interacting trafficking regulators, including ARF4 and VCP: ARF4 enhances NIS trafficking from the Golgi to the plasma membrane, whereas VCP promotes NIS proteolysis [45]. Oncogenic transformation can also contribute to this phenotype. In papillary thyroid carcinoma, both BRAF-mutant and BRAF-wild-type tumors may show predominantly cytoplasmic NIS staining [9], while BRAFV600E-driven models further support the broader role of oncogenic signaling in suppressing functional NIS expression and radioiodine avidity [10,44].

The clinical interpretation of NIS localization by immunohistochemistry requires caution. Subcellular localization is biologically relevant because only membrane-localized NIS can mediate iodide uptake, and predominantly cytoplasmic staining is consistent with impaired transporter function [9,10,38]. However, the attached sources do not justify using NIS localization alone as a robust standalone predictor of successful ablation or disease control. Moreover, later immunoanalysis challenged the earlier interpretation of “intracellular hyperexpression,” reporting that much of the intracellular staining obtained with anti-NIS antibodies may reflect non-specific signal and that increasing NIS expression, not merely improving trafficking, remains an important therapeutic goal [47]. Accordingly, cytoplasmic staining should be interpreted as supportive rather than definitive evidence of functional NIS loss, ideally together with radioiodine imaging and other clinicopathological data.

Understanding the multilevel regulation of NIS expression and localization opens opportunities for developing targeted therapeutic strategies aimed at restoring symporter functional activity. MAPK-pathway inhibitors and other redifferentiation approaches may partially restore NIS expression and radioiodine uptake in selected settings [2,10,44], while agents targeting trafficking regulators have shown preclinical promise. In particular, VCP inhibitors increased cell-surface NIS and enhanced radioiodine uptake in mouse and human thyroid models [45]. These findings support NIS trafficking as a therapeutically relevant target, although the current evidence remains predominantly preclinical.

### 4.3. Tumor Dedifferentiation and Transcription Factor Suppression

Tumor cell dedifferentiation is a key biological process underlying the development of radioiodine refractoriness in differentiated thyroid cancer. This process is characterized by loss of thyroid-specific functional properties, including expression of thyroglobulin, thyroid peroxidase, and, most importantly for radioiodine therapy, the sodium/iodide symporter (NIS) [1,2,8]. At the molecular level, dedifferentiation reflects disruption of the thyroid-specific transcriptional program that is normally maintained by lineage-defining factors, particularly PAX8 and NKX2-1, with additional contributions from other thyroid transcription factors such as FOXE1 [13,14].

PAX8 has a central role in thyroid follicular-cell differentiation. Pax8-deficient mice fail to form thyroid follicular cells, indicating that Pax8 is required for commitment of competent endodermal primordia to the thyroxin-producing follicular lineage [48,49]. In differentiated thyrocytes, PAX8 participates directly in transcriptional control of thyroid-specific genes, including SLC5A5/NIS, through the NIS upstream enhancer [42,43].

NKX2-1 (TTF-1, TITF-1) is another major regulator of thyroid differentiation. Nkx2-1/Ttf1-null mice lack the thyroid gland, demonstrating that this transcription factor is essential for thyroid organogenesis [49]. In functional studies, NKX2-1 binds the rat NIS promoter and increases its transcriptional activity, supporting a direct role in the maintenance of iodide-transport machinery [50]. More broadly, thyroid-specific transcription relies on a combinatorial network rather than on any single factor alone, and the thyroid-restricted co-expression of PAX8 and NKX2-1 is a recurring feature of the differentiated thyrocyte phenotype [13,50,51].

#### 4.3.1. Transcription Factor Suppression During DTC Progression

During thyroid tumor progression, the differentiated transcriptional program becomes increasingly unstable. Experimental and pathological data indicate that expression of NKX2-1 is usually retained in differentiated papillary and follicular carcinomas, whereas it is lost in anaplastic thyroid cancers, consistent with the near-complete collapse of the follicular-cell phenotype at that stage [13]. In parallel, experimental reintroduction of Pax8 into transformed thyroid cells is sufficient to restore endogenous expression of thyroglobulin, thyroperoxidase, and NIS, showing that impairment of this regulatory axis can directly contribute to loss of differentiation [14].

Current evidence also indicates that dedifferentiation cannot be explained simply by the uniform disappearance of a single transcription factor. In papillary thyroid carcinomas, BRAF-mutant tumors show markedly reduced expression of NIS, thyroglobulin, thyroperoxidase, and other iodine-handling genes, whereas PAX8 expression may remain comparably reduced in both BRAF-mutant and BRAF-wild-type tumors [9]. This pattern suggests that oncogenic signaling can suppress the thyroid differentiation program at multiple levels downstream of, or in parallel with, lineage transcription factors.

Mechanistically, oncogenic MAPK signaling is particularly important. In BRAFV600E-positive models, constitutive pathway activation induces an autocrine TGF-β/SMAD loop that represses NIS expression while promoting epithelial–mesenchymal transition, migration, and invasion [44]. Together, these findings support a model in which tumor progression involves both disturbance of lineage transcription-factor networks and direct repression of thyroid-specific effector genes by oncogenic signaling pathways [2,9,13,44].

#### 4.3.2. Dedifferentiation and Loss of Iodine Uptake

Disruption of the PAX8/NKX2-1-centered differentiation network has direct consequences for iodide handling because NIS is one of its downstream effector genes. Pax8 binds the NIS upstream enhancer, and NKX2-1 activates the NIS promoter, providing a mechanistic basis for the dependence of iodide uptake on preservation of thyroid lineage transcription [42,43,50]. When this program is impaired, expression of NIS falls together with other markers of thyroid differentiation such as thyroglobulin and thyroperoxidase [2,9,13].

The functional consequence is loss of the coordinated machinery required for radioiodine uptake and retention. This is particularly evident in BRAF-mutant papillary thyroid carcinomas, in which expression of NIS and other iodine-metabolism genes is lower than in BRAF-wild-type tumors, while the residual NIS protein is frequently mislocalized to the cytoplasm rather than the plasma membrane [9]. Thus, dedifferentiation reduces radioiodine avidity not only by decreasing NIS abundance, but also by disrupting the broader thyroid-specific gene program required for effective iodide transport and organification [2,8,9].

Clinically, this framework supports the close association between preservation of thyroid differentiation and retained radioiodine responsiveness in metastatic DTC, whereas tumors that lose thyroid-specific gene expression are more likely to become radioiodine refractory [1,2,8]. However, the attached sources do not justify treating isolated PAX8 or NKX2-1 expression levels as standalone clinical predictors; dedifferentiation is better interpreted as a multilevel process integrating lineage transcription factor, downstream effector genes, oncogenic signaling, and NIS subcellular localization [2,9,13,44].

### 4.4. Post-Transcriptional Regulation Through MicroRNAs

MicroRNAs (miRNAs) are small non-coding RNA molecules (~22 nucleotides) that regulate gene expression post-transcriptionally by binding to complementary sequences in the 3′-untranslated region (3′-UTR) of target mRNAs, leading to translational repression or mRNA degradation [17]. In differentiated thyroid cancer, dysregulated miRNA expression contributes to tumor progression and may influence radioiodine response through effects on proliferation, invasion, lineage-associated pathways, and, in some cases, direct repression of genes relevant to iodide handling [17,18,19,20,21,22].

#### 4.4.1. Oncogenic MicroRNAs: miR-221-3p and miR-222-3p

MicroRNAs miR-221-3p and miR-222-3p are encoded by a cluster on chromosome X and are frequently co-expressed in papillary thyroid carcinoma [19]. Both miR-221-3p and miR-222-3p are significantly overexpressed in thyroid papillary carcinomas compared to normal thyroid tissue [19]. These microRNAs exert oncogenic effects through direct targeting of p27Kip1 (CDKN1B), a cyclin-dependent kinase inhibitor, leading to accelerated cell cycle progression and increased proliferation [19].

The relevance of miR-221-3p/miR-222-3p to radioiodine refractoriness is currently best supported indirectly. In papillary thyroid cancer models, HMGB1-induced overexpression of miR-221-3p and miR-222-3p increases tumor-cell growth and motility, linking this oncogenic cluster to a more aggressive phenotype [20]. However, the attached sources do not directly demonstrate that miR-221-3p targets PAX8 or that miR-222-3p targets NKX2-1. Accordingly, these microRNAs are better interpreted as contributors to tumor progression and dedifferentiation-associated behavior rather than as established direct repressors of the thyroid transcription-factor network [19,20].

#### 4.4.2. Tumor-Suppressor MicroRNA: miR-204-5p

MicroRNA-204-5p functions as a tumor suppressor with downregulated expression in papillary thyroid carcinoma [21,22]. One experimentally validated mechanism involves direct targeting of HMGA2 (high mobility group AT-hook 2), an oncogenic chromatin-associated factor [21]. In thyroid cancer cells, miR-204-5p overexpression suppresses HMGA2 and inhibits proliferation [21].

Additional experimentally supported targets include IGFBP5 (insulin-like growth factor-binding protein 5), whose repression contributes to reduced proliferation and increased apoptosis in papillary thyroid carcinoma cells [22]. Taken together, these studies support miR-204-5p as a tumor-suppressive miRNA in PTC. However, the attached sources do not directly support the stronger claim that miR-204-5p stabilizes FOXE1 or restores NIS expression through an HMGA2/FOXE1 axis.

#### 4.4.3. Oncogenic MicroRNA: miR-146b-3p

MicroRNA-146b-3p is among the most consistently overexpressed microRNAs in papillary thyroid carcinoma and has been proposed as both a diagnostic and prognostic biomarker [18]. Experimental studies summarized in the attached review indicate that miR-146b-3p can directly target PAX8 and NIS, thereby reducing iodide-handling capacity and radioactive iodide sensitivity in thyroid cancer models [18]. This makes miR-146b-3p the clearest miRNA in the present section with a direct mechanistic link to the PAX8/NIS axis.

In addition, miR-146b-3p has been implicated in aggressive tumor behavior through regulation of pathways involving TGF-beta resistance, EMT, migration, invasion, and cytoskeletal remodeling [18]. High miR-146b-3p expression is associated with extrathyroidal extension, lymph node metastasis, BRAFV600E mutation status, and increased risk of recurrence in papillary thyroid carcinoma [18]. By contrast, the attached sources do not support extending this direct mechanistic interpretation to miR-221-3p or miR-222-3p at the same level of evidence.

#### 4.4.4. Clinical Significance and Prognostic Value

The strongest current clinical evidence in the attached sources concerns prognosis after treatment rather than strict definition of radioiodine-refractory disease. A propensity-score matched analysis showed that a microRNA-based score combining high miR-221-3p/miR-222-3p with low miR-204-5p predicts tumor progression after postoperative radioactive iodine ablation in well-differentiated thyroid cancer [23]. Patients with high-risk scores had substantially shorter progression-free survival, and the score outperformed ATA risk stratification in that cohort [23]. These data support prognostic value after RAI treatment, but they should not be equated automatically with formal ATA-defined radioiodine refractoriness.

Separately, circulating serum miR-146a-5p and miR-221-3p have shown potential as non-invasive biomarkers for papillary thyroid carcinoma diagnosis and for monitoring disease progression during follow-up using digital PCR [52]. In that study, changes in fold expression, rather than absolute concentrations alone, helped identify progressive disease even when thyroglobulin was uninformative [52].

The candidate biomarker landscape likely extends beyond the most intensively studied microRNAs addressed above. For example, miR-125b has been linked in papillary thyroid cancer to autophagy-related signaling, BRAFV600E status, invasion, and recurrence risk, supporting broader evaluation of multi-miRNA biomarker panels in future translational studies [53].

Table 1 summarizes the key microRNAs discussed here and the level of mechanistic support currently available for their association with thyroid differentiation-associated pathways and radioiodine response [18,19,20,21,22,23].

Importantly, approximately 30–40% of differentiated thyroid cancers lack identifiable BRAF, RAS mutations or RET/NTRK fusions [15], suggesting that microRNA dysregulation may represent a key alternative mechanism of radioiodine resistance. Integration of microRNA expression profiling with genetic biomarkers (BRAF, TERT promoter status) and epigenetic markers (SLC5A5 promoter methylation) may enable comprehensive molecular characterization for personalized risk stratification and treatment approaches [2,23].

### 4.5. Genetic Factors of Radioiodine Resistance

Genetic changes underlying tumor transformation in differentiated thyroid cancer play a central role in radioiodine refractoriness development. Somatic mutations in key oncogenes and tumor suppressors lead to constitutive activation of intracellular signaling cascades that suppress thyroid differentiation and sodium/iodide symporter expression. Identification of specific genetic alterations associated with loss of iodine uptake not only improves understanding of radioiodine resistance molecular mechanisms but also opens possibilities for personalized therapeutic approaches aimed at restoring tumor radioactive iodine sensitivity [1,2,9,10,11,12,15].

#### 4.5.1. BRAF V600E Mutation and MAPK Pathway Activation

The somatic BRAF V600E mutation is the most frequent genetic event in papillary thyroid cancer, occurring in 40–45% of cases [1,9,15]. This point mutation leads to substitution of valine with glutamic acid at position 600 of the b-RAF protein, causing constitutive activation of its kinase activity independent of upstream signals [9,15]. The BRAF V600E mutation is a critical molecular event providing constitutive activation of the MAPK/ERK signaling pathway in thyroid cancer [16]. Activated BRAF-V600E constitutively phosphorylates and activates MEK1/2, which in turn phosphorylate ERK1/2, leading to uncontrolled activation of the entire MAPK (mitogen-activated protein kinases) cascade [9,15,16].

MAPK pathway activation exerts multiple inhibitory effects on NIS expression and function [9,44]. Molecular studies demonstrate that thyroid cancer cells with the BRAF V600E mutation are characterized by significantly lower NIS mRNA and protein levels compared to BRAF-negative tumors [9,16,44]. Mechanisms of NIS suppression during MAPK pathway activation include induction of transforming growth factor beta (TGF-β) secretion, which directly represses the SLC5A5 gene promoter via SMAD-dependent signaling, as well as additional alterations in thyroid differentiation programs and chromatin regulation described in other models [16,44].

Clinical data convincingly demonstrate the association of BRAF V600E mutation with radioiodine refractoriness and worse prognosis [1,9,15]. Meta-analyses show that BRAF-positive tumors demonstrate clinical radioiodine therapy refractoriness 2–3 times more frequently compared to BRAF-negative carcinomas [15]. Patients with BRAF V600E mutation have increased risk of disease recurrence, distant metastases development, and tumor-specific mortality [1,15]. Critically, the association of BRAF status with radioiodine refractoriness is not absolute: not all BRAF-positive tumors are refractory, and radioiodine resistance can develop in BRAF-negative status, indicating the existence of alternative mechanisms [1,2,9].

Understanding the central role of activated MAPK pathway in NIS suppression served as the basis for developing redifferentiation therapy using BRAF and MEK inhibitors [2,15]. A clinical study with selumetinib (MEK inhibitor) demonstrated the ability to restore radioiodine uptake in 12 of 20 evaluable patients with previously refractory DTC, with eight patients receiving RAI treatment and five achieving partial response [54]. Subsequent studies with dabrafenib (BRAF V600E inhibitor) further confirmed the potential of MAPK pathway inhibition to restore iodine uptake in refractory BRAF-mutant tumors [55].

#### 4.5.2. TERT Promoter Mutations

Mutations in the TERT (telomerase reverse transcriptase) gene promoter represent the second most frequent genetic change in aggressive forms of differentiated thyroid cancer [1]. Somatic mutations in the TERT gene promoter region, first discovered in melanoma, create new binding sites for ETS transcription factors and lead to telomerase activation [56]. These mutations were subsequently shown to have high prevalence in aggressive forms of thyroid cancer [57]. These mutations, most commonly represented by C228T (chr5:1,295,228 C > T) and C250T (chr5:1,295,250 C > T) substitutions, create new binding sites for ETS family transcription factors, leading to increased TERT promoter transcriptional activity and telomerase hyperexpression [1,56,57]. TERT promoter mutation frequency increases with tumor aggressiveness: from 10 to 15% in typical papillary carcinomas to 30–40% in poorly differentiated forms and up to 70% in anaplastic thyroid cancer [57].

TERT mutations are associated with significantly worse prognosis and increased radioiodine refractoriness risk [1,57]. TERT-mutant thyroid tumors are characterized by a more aggressive clinical course, increased frequency of extrathyroidal invasion, distant metastases, and tumor-specific mortality [57]. Patients with TERT-mutant tumors have increased frequency of distant metastases, recurrences, and tumor-specific mortality [1,57]. A particularly unfavorable situation is observed with the combination of BRAF V600E and TERT promoter mutations: such tumors are characterized by an extremely aggressive clinical course, high radioiodine refractoriness frequency, and poor overall prognosis [1,57]. The combination of these mutations has a synergistic effect in suppressing differentiation and promoting tumor progression [57].

Molecular mechanisms linking TERT mutations with radioiodine resistance are not fully established but likely include telomere stabilization providing unlimited replicative potential of tumor cells, facilitating accumulation of additional genetic and epigenetic changes leading to dedifferentiation [1]. TERT hyperexpression may also exert direct non-canonical effects on cellular signaling pathways affecting differentiation and NIS expression [1].

#### 4.5.3. MAPK and PI3K/AKT Pathway Coactivation

Besides BRAF mutations, a significant portion of differentiated thyroid carcinomas is characterized by PI3K/AKT pathway activation, often combined with MAPK cascade activation [2,15]. PI3K/AKT activation may be driven by RAS-family mutations, activating alterations of the PI3K catalytic subunit (PIK3CA), or inactivating changes affecting the PTEN tumor suppressor [2,12]. RAS mutations lead to simultaneous activation of both MAPK and PI3K/AKT pathways, creating a powerful oncogenic signal [2].

Both MAPK and PI3K/AKT pathways converge on the suppression of thyroid differentiation and NIS expression through complementary mechanisms [2,9,44]. The MAPK cascade induces TGF-β secretion that directly represses the SLC5A5 promoter through SMAD-dependent signaling and promotes a more invasive, dedifferentiated phenotype [44]. BRAF mutations inhibit expression of iodine metabolism genes including NIS, AIT, TPO, and TG [9]. BRAFV600E induces TGF-β secretion, which represses NIS through SMAD-dependent signaling [44]. The PI3K/AKT pathway exerts parallel inhibitory effects by reducing iodide-metabolism gene expression, activating mTOR-dependent suppressive signaling, and impairing NIS-mediated iodide transport [2].

Coactivation of both pathways exerts strong suppressive effects on NIS expression and can contribute to radioiodine refractoriness development [2]. Tumors with simultaneous activation of these signaling networks tend to show deeper dedifferentiation and greater biological aggressiveness [2]. These observations have critical therapeutic implications: monotherapy with a single pathway inhibitor may be insufficient for restoring iodine uptake when multiple cascades are coactivated [2]. Combination strategies targeting pathway feedback and complementary resistance mechanisms have shown more pronounced redifferentiating effects in preclinical models and may represent a promising approach for tumors with complex molecular profiles [2,11,15].

Additional genetic factors contributing to radioiodine resistance include inactivating TP53 gene mutations, activating β-catenin (CTNNB1) mutations, and alterations in chromatin-remodeling complex genes [1,15]. TP53 mutations are particularly characteristic of poorly differentiated and anaplastic carcinomas and are associated with extremely aggressive course and absolute radioiodine refractoriness [1]. Integration of these multiple genetic alterations into prognostic models allows more accurate prediction of radioiodine resistance development risk and personalization of therapeutic approaches [1,15].

Thus, genetic factors play a central role in determining thyroid tumor radioiodine status. BRAF V600E mutations, TERT promoter mutations, MAPK and PI3K/AKT pathway activation through various genetic mechanisms converge on thyroid differentiation and NIS expression suppression. Tumor molecular profiling, including assessment of BRAF, RAS, TERT, and other key genetic alterations, is increasingly relevant to clinical management of differentiated thyroid cancer, particularly for risk stratification, selection of targeted therapies, and interpretation of follicular and oncocytic tumor biology in molecular testing workflows [1,2,15,58].

### 4.6. Epigenetic NIS Suppression

Epigenetic gene expression regulation represents a fundamental mechanism of cellular differentiation and function control, acting without changing the primary DNA sequence. In the context of differentiated thyroid cancer radioiodine refractoriness, epigenetic suppression of the SLC5A5 gene encoding the sodium/iodide symporter plays a key role alongside genetic and transcriptional mechanisms. Hypermethylation of NIS gene promoter and enhancer regions, as well as repressive histone modifications, lead to stable transcriptional gene silencing even under conditions of preserved differentiation transcription factor activity [2,8,59,60]. Understanding NIS suppression epigenetic mechanisms opens possibilities for pharmacological gene reactivation using demethylating agents and histone deacetylase inhibitors, which forms the basis of redifferentiation therapy strategies [2,59,61].

#### 4.6.1. DNA Methylation of SLC5A5 Gene Promoter and Enhancer

Methylation of cytosine residues in CpG dinucleotides of gene regulatory regions is a classic epigenetic silencing mechanism, leading to long-term transcription suppression [59,60]. The human SLC5A5 gene contains a CpG-rich proximal promoter region, and later studies additionally identified distal regulatory elements (enhancers) sensitive to epigenetic modification [59,60]. In paired thyroid samples, lower methylation in surrounding non-tumor tissue and higher methylation in tumors have been associated with reduced NIS expression [60].

Pioneering studies showed that 5-azacytidine can restore hNIS mRNA expression in several dedifferentiated thyroid cell lines and recover iodide transport in a subset of them, supporting a role for DNA methylation in NIS gene suppression [59]. That study also found that promoter-to-first-intron methylation patterns in human tumor samples were heterogeneous and not uniformly predictive of transcriptional failure [59]. Subsequent work identified the distal enhancer as an additional epigenetically regulated element associated with lower NIS mRNA and protein expression in thyroid tumors [60].

An important recent discovery was the identification of a new NIS distal enhancer (NDE), located at positions −2152/−1887 relative to the ATG start codon [60]. In paired thyroid samples, hypermethylation of this enhancer showed a significant inverse correlation with NIS mRNA expression and was detected in both benign and malignant tumors compared with surrounding non-tumor tissue [60]. Functional assays demonstrated enhancer activity of this region, which was most evident in the presence of PAX8 and NKX2.1, although canonical binding sites for these factors were not directly identified [60]. These data indicate that epigenetic dysregulation of distal SLC5A5 regulatory elements can contribute to reduced NIS expression independently of proximal promoter methylation alone [60].

#### 4.6.2. Histone Modifications and Chromatin Architecture

Besides DNA methylation, an important role in epigenetic regulation is played by post-translational modifications of histone proteins constituting the nucleosome core chromatin [62]. Acetylation of histones H3 and H4, catalyzed by histone acetyltransferases (HAT), is associated with open chromatin conformation and active transcription, whereas histone deacetylation by histone deacetylases (HDAC) leads to chromatin compaction and transcriptional repression [62]. In thyroid tumors, global histone acetylation patterns are altered: H3K9-K14ac and H3K18ac are increased in differentiated tumors relative to normal tissue, whereas the H3K18ac pattern changes with progression to undifferentiated carcinoma [62].

Given the reversibility of histone acetylation and the altered acetylation landscape in thyroid tumors [62], HDAC inhibition by pharmacological agents (valproic acid, vorinostat, romidepsin) has been explored as a strategy to promote a more permissive chromatin state and, in some cases, restore radioiodine avidity [2,61]. Because epigenetic silencing involves multiple regulatory layers, the combined application of demethylating agents and HDAC inhibitors has been proposed as a rational approach to NIS reactivation rather than monotherapy with either drug class alone [2].

#### 4.6.3. Mechanisms of NIS Epigenetic Silencing Induction

Epigenetic NIS suppression in thyroid cancer is not a random event but can be reinforced by oncogenic signaling [2,9]. MAPK pathway activation in BRAF V600E or RAS-mutant tumors contributes to loss of differentiation, while BRAF-driven TGF-β/SMAD signaling provides an additional mechanism of NIS repression [44].

Transforming growth factor beta (TGF-β), whose secretion is induced by activated BRAFV600E signaling, contributes to transcriptional repression of NIS through SMAD-dependent pathway activation [44].

#### 4.6.4. Demethylating and Redifferentiation Therapy

The reversibility of epigenetic modifications, unlike genetic mutations, opens unique therapeutic opportunities for restoring NIS expression and overcoming radioiodine refractoriness [2,60]. In preclinical thyroid models, treatment with 5-aza-2′-deoxycytidine reduced NDE methylation concomitantly with restoration of NIS mRNA and protein expression and increased 125I uptake [60]. Histone deacetylase (HDAC) inhibitors, including valproic acid, vorinostat, panobinostat, and romidepsin, prevent histone deacetylation and can facilitate a more permissive chromatin state [2,61,62].

Preclinical studies on demethylating therapy demonstrate restoration of NIS mRNA expression and functional iodine uptake, and in some models also restoration of NIS protein expression, in thyroid cancer models [59,60]. A clinical study using romidepsin (HDAC inhibitor) documented restoration of RAI avidity in two patients, but no major RECIST responses were observed and the overall clinical benefit was limited [61]. Review and preclinical data suggest that combination of epigenetic modulators with oncogenic kinase inhibitors (MEK, BRAF) may be more effective, simultaneously targeting both transcriptional and epigenetic barriers to NIS expression [2].

#### 4.6.5. Epigenetic Biomarkers of Radioiodine Refractoriness

Assessment of SLC5A5 promoter and enhancer methylation, together with other molecular data, may be explored as a candidate biomarker framework for radioiodine refractoriness [2]. Methylation of SLC5A5 regulatory regions is mechanistically associated with reduced NIS expression and impaired iodine uptake in experimental models and in paired tumor-versus-nontumor analyses [59,60]. Conversely, low NIS expression in the absence of substantial methylation suggests that transcriptional or signaling-mediated suppression may predominate [2]. Integration of epigenetic biomarkers into comprehensive prognostic panels together with genetic and expression data may allow more accurate patient stratification and personalized therapeutic approaches [2].

### 4.7. Redifferentiation Therapy: Restoration of Iodine Uptake

Understanding the key role of dedifferentiation in radioiodine refractoriness development served as the basis for developing redifferentiation therapy strategies—an approach aimed at pharmacological restoration of thyroid differentiation programs and, in some tumors, recovery of NIS expression, localization, and radioactive iodine uptake [2,15,63]. The best-characterized clinical example of this strategy is the use of MAPK pathway inhibitors, particularly selumetinib (MEK inhibitor), in patients with BRAF- or RAS-mutant radioiodine-refractory DTC [54,55].

A pioneering clinical trial demonstrated the principal possibility of redifferentiation with selumetinib application [54]. In 12 of 20 evaluable patients with radioiodine-refractory DTC, selumetinib increased iodine-124 uptake on repeat PET/CT, and eight of these patients met the prespecified dosimetric threshold for therapeutic radioiodine administration [54]. Among the eight patients who received iodine-131, five had confirmed partial responses and three had stable disease, while all showed marked decreases in serum thyroglobulin levels [54].

Subsequent studies demonstrated successful iodine uptake restoration using dabrafenib—a specific BRAFV600E mutant kinase inhibitor—in patients with BRAF-mutant radioiodine-refractory papillary thyroid cancer [55]. After 25 days of dabrafenib treatment, 6 of 10 patients showed new RAI uptake and continued therapy before receiving therapeutic I-131; at three-month restaging, two achieved partial response and four had stable disease [55]. This study supports direct oncogenic driver inhibition as an effective redifferentiation strategy in BRAF-mutant disease [55].

However, the phase III randomized placebo-controlled ASTRA trial evaluated selumetinib in a different clinical context—the adjuvant setting in patients with high-risk DTC (primary tumor > 4 cm, gross extrathyroidal extension, or significant lymph node involvement) [64]. In this trial, 233 patients were randomized 2:1 to receive selumetinib or placebo for approximately 5 weeks before adjuvant RAI administration. No statistically significant difference in complete remission rate at 18 months was observed (selumetinib 40% vs. placebo 38%; OR 1.07, *p* = 0.82) [64]. The ASTRA results show that adding selumetinib to adjuvant RAI did not improve remission rates in unselected high-risk patients in the postoperative setting. These findings support a more selective strategy for redifferentiation approaches, with emphasis on tumor genotype-tailored drug selection and maintenance of adequate dosing to optimize RAI efficacy [63,64].

Additional redifferentiation therapy strategies include epigenetic modulator application—demethylating agents and histone deacetylase (HDAC) inhibitors, which can reactivate differentiation gene expression through epigenetic remodeling [2,59]. Although clinical study results with these agents are less impressive compared to kinase inhibitors, combined approaches combining epigenetic modulators with signaling pathway inhibitors represent a promising direction for overcoming radioiodine refractoriness [2,15].

Thus, tumor cell dedifferentiation, involving disruption of lineage transcription-factor networks together with repression of downstream iodine-handling genes, represents a central mechanism of radioiodine refractoriness development in DTC. Understanding the molecular basis of this process not only explains the clinical phenomenon of iodine uptake loss during disease progression, but also opens possibilities for redifferentiation therapy aimed at restoring radioactive iodine sensitivity in selected patients.

## 5. Conclusions

NIS downregulation is the central molecular determinant of radioiodine refractoriness in differentiated thyroid cancer. This review demonstrates that NIS loss results from multilevel dysregulation: transcriptional suppression through oncogenic MAPK/ERK and PI3K/AKT pathway activation that disrupts thyroid differentiation programs involving PAX8, NKX2-1, and FOXE1; impaired post-translational trafficking with cytoplasmic sequestration of NIS protein; epigenetic silencing via SLC5A5 promoter and enhancer hypermethylation and repressive histone modifications; and post-transcriptional regulation by oncogenic microRNAs. Genetic alterations, particularly BRAFV600E and TERT promoter mutations, are associated with dedifferentiated tumor phenotypes and poor radioiodine response [1,2,10].

MicroRNA dysregulation represents a key complementary mechanism, especially in tumors without clearly identifiable canonical driver alterations. The oncogenic miR-221-3p/miR-222-3p cluster is consistently associated with proliferation and aggressive tumor behavior, miR-204-5p shows tumor-suppressive effects through targets such as HMGA2 and IGFBP5, and miR-146b-3p has the strongest direct experimental link to impaired thyroid differentiation and reduced radioactive iodide sensitivity [17,18,19,20,21,22]. The available clinical data support microRNA panels as prognostic and monitoring tools after treatment rather than as standalone formal definitions of radioiodine refractoriness [23], while circulating miR-146a-5p and miR-221-3p show promise for PTC diagnosis and disease monitoring [52].

Redifferentiation therapy using MAPK pathway inhibitors (selumetinib, dabrafenib) has demonstrated the feasibility of restoring iodine uptake in selected patients with RAI-refractory disease, validating NIS as a therapeutic target [54,55], although the phase III ASTRA trial showed no benefit of selumetinib in the adjuvant setting [64]. Novel approaches targeting NIS trafficking, including VCP inhibitors that enhance NIS membrane localization and radioiodine uptake in preclinical models, represent promising complementary strategies [45]. Integration of genetic (BRAF, TERT), epigenetic (SLC5A5 methylation), and microRNA biomarkers into comprehensive prognostic panels—together with multivariate prediction models incorporating clinicopathological, serological, and imaging parameters [41]—may enable personalized risk stratification and treatment selection. Future research should focus on standardization of microRNA-based diagnostic assays and prospective validation of combined biomarker panels, while redifferentiation trials require more selective, genotype-tailored strategies and standardized protocols to define which patients are most likely to benefit [63].

## Figures and Tables

**Table 1:** Key microRNAs associated with thyroid cancer progression, differentiation-associated pathways, and radioiodine response in differentiated thyroid cancer [18,19,20,21,22,23].

MicroRNA	Functional Class	Main Supported Target(s)	Expression Pattern	Evidence Relevant to Radioiodine Response
miR-221-3p	Oncogenic	p27Kip1/CDKN1B	Upregulated	Indirect relevance through increased proliferation and, with HMGB1 stimulation, enhanced tumor-cell growth and motility
miR-222-3p	Oncogenic	p27Kip1/CDKN1B	Upregulated	Indirect relevance through increased proliferation and, with HMGB1 stimulation, enhanced tumor-cell growth and motility
miR-146b-3p	Oncogenic	PAX8 and NIS	Upregulated	Direct mechanistic link to impaired iodide handling and reduced radioactive iodide sensitivity
miR-204-5p	Tumor-suppressor	HMGA2; IGFBP5	Downregulated	Indirect relevance through tumor-suppressive effects; attached sources do not directly establish FOXE1/NIS restoration

## Data Availability

No new data were created or analyzed in this study. Data sharing is not applicable to this article.

## Abbreviations

The following abbreviations are used in this manuscript:

AKT	protein kinase B
ARF4	ADP-ribosylation factor 4
ATA	thyroid-stimulating hormone
BRAF	B-Raf proto-oncogene, serine/threonine kinase
cAMP	cyclic adenosine monophosphate
CDKN1B	cyclin-dependent kinase inhibitor 1B (p27Kip1)
CT	computed tomography
ctDNA	circulating tumor DNA
CTNNB1	catenin beta-1 (β-catenin)
DNMT	DNA methyltransferase
DTC	differentiated thyroid cancer
DUOX2	dual oxidase 2
EMT	epithelial–mesenchymal transition
ERK	extracellular signal-regulated kinase
ETS	E26 transformation-specific (transcription factor family)
FDG	fluorodeoxyglucose
FOXE1	forkhead box E1
FTC	follicular thyroid carcinoma
HAT	histone acetyltransferase
HDAC	histone deacetylase
HMGA2	high mobility group AT-hook 2
IGF-1	insulin-like growth factor-1
IGFBP5	insulin-like growth factor-binding protein 5
MAPK	mitogen-activated protein kinase
MeCP2	methyl-CpG binding protein 2
MEK	mitogen-activated protein kinase kinase
miRNA (miR)	microRNA
MRI	magnetic resonance imaging
mTOR	mechanistic target of rapamycin
NDE	NIS distal enhancer
NF-κB	nuclear factor kappa-light-chain-enhancer of activated B cells
NIS	sodium/iodide symporter
NKX2-1 (TTF-1)	NK2 homeobox 1 (thyroid transcription factor-1)
PAX8	paired box gene 8
PI3K	phosphoinositide 3-kinase
PIK3CA	phosphatidylinositol-4,5-bisphosphate 3-kinase catalytic subunit alpha
PKA	protein kinase A
PET/CT	positron emission tomography/computed tomography
PFS	progression-free survival
PTC	papillary thyroid carcinoma
PTEN	phosphatase and tensin homolog
RAI	radioiodine therapy
RAIR	radioiodine-refractory
RAS	rat sarcoma viral oncogene homolog
RECIST	Response Evaluation Criteria in Solid Tumors
RR-DTC	radioiodine-refractory differentiated thyroid cancer
SLC5A5	solute carrier family 5 member 5 (NIS gene)
SPECT/CT	single-photon emission computed tomography/computed tomography
TERT	telomerase reverse transcriptase
TG	thyroglobulin
TGF-β	transforming growth factor beta
TPO	thyroid peroxidase
TSH	thyroid-stimulating hormone
TSHR	thyroid-stimulating hormone receptor
VCP	valosin-containing protein
WBS	whole-body scintigraphy
